# Supplementary material for: Causal Relationship between Aging and Anorexia Nervosa: A White-Matter-Microstructure-Mediated Mendelian Randomization Analysis
Source: Biomedicines. 2024 Aug 16;12(8):1874. doi: 10.3390/biomedicines12081874 (PMC11351342; doi:10.3390/biomedicines12081874)
Supplement: Supplementary file 1 [file biomedicines-12-01874-s001.zip › Supplementary Figure/Figure S4/Singe SNP plot of MR analysis for TL to ubm-b-1657.pdf]

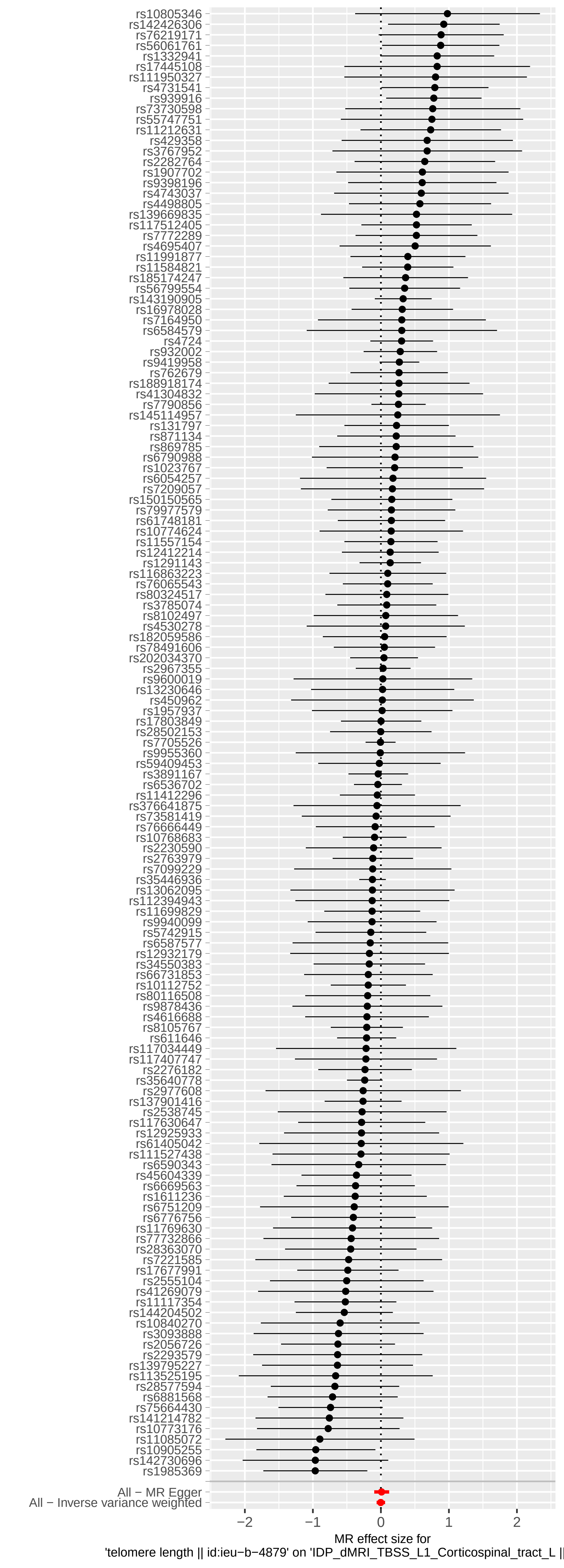

rs10805346
rs142426306
rs76219171
rs56061761
rs1332941
rs17445108
rs111950327
rs4731541
rs939916
rs73730598
rs55747751
rs11212631
rs429358
rs3767952
rs2282764
rs1907702
rs9398196
rs4743037
rs4498805
rs139669835
rs117512405
rs7772289
rs4695407
rs11991877
rs11584821
rs185174247
rs56799554
rs143190905
rs16978028
rs7164950
rs6584579
rs4724
rs932002
rs9419958
rs762679
rs188918174
rs41304832
rs7790856
rs145114957
rs131797
rs871134
rs869785
rs6790988
rs1023767
rs6054257
rs7209057
rs150150565
rs79977579
rs61748181
rs10774624
rs11557154
rs12412214
rs1291143
rs116863223
rs76065543
rs80324517
rs3785074
rs8102497
rs4530278
rs182059586
rs78491606
rs202034370
rs2967355
rs9600019
rs13230646
rs450962
rs1957937
rs17803849
rs28502153
rs7705526
rs9955360
rs59409453
rs3891167
rs6536702
rs11412296
rs376641875
rs73581419
rs76666449
rs10768683
rs2230590
rs2763979
rs7099229
rs35446936
rs13062095
rs112394943
rs11699829
rs9940099
rs5742915
rs6587577
rs12932179
rs34550383
rs66731853
rs10112752
rs80116508
rs9878436
rs4616688
rs8105767
rs611646
rs117034449
rs117407747
rs2276182
rs35640778
rs2977608
rs137901416
rs2538745
rs117630647
rs12925933
rs61405042
rs111527438
rs6590343
rs45604339
rs6669563
rs1611236
rs6751209
rs6776756
rs11769630
rs77732866
rs28363070
rs7221585
rs17677991
rs2555104
rs41269079
rs11117354
rs144204502
rs10840270
rs3093888
rs2056726
rs2293579
rs139795227
rs113525195
rs28577594
rs6881568
rs75664430
rs141214782
rs10773176
rs11085072
rs10905255
rs142730696
rs1985369
All − MR Egger
All − Inverse variance weighted
−2
−1
0
1
2
MR effect size for
'telomere length || id:ieu−b−4879' on 'IDP_dMRI_TBSS_L1_Corticospinal_tract_L ||
